# Sulfur resistance of Ce-Mn/TiO_2_ catalysts for low-temperature NH_3_–SCR

**DOI:** 10.1098/rsos.171846

**Published:** 2018-03-07

**Authors:** Quan Xu, Wenjing Yang, Shitong Cui, Jason Street, Yan Luo

**Affiliations:** 1 State Key Laboratory of Heavy Oil Processing, Institute of New Energy, China University of Petroleum, Beijing 102249, People's Republic of China; 2 Department of Sustainable Bioproducts, Mississippi State University, Mississippi, MS 39762, USA; 3 Department of Chemical Engineering, West Virginia University, Morgantown, WV 26505, USA

**Keywords:** NH_3_–SCR, sulfur resistance, Ce-Mn/TiO_2_ catalyst, catalyst synthesis

## Abstract

Ce-Mn/TiO_2_ catalyst prepared using a simple impregnation method demonstrated a better low-temperature selective catalytic reduction of NO with NH_3_ (NH_3_–SCR) activity in comparison with the sol-gel method. The Ce-Mn/TiO_2_ catalyst loading with 20% Ce had the best low-temperature activity and achieved a NO conversion rate higher than 90% at 140–260°C with a 99.7% NO conversion rate at 180°C. The Ce-Mn/TiO_2_ catalyst only had a 6% NO conversion rate decrease after 100 ppm of SO_2_ was added to the stream. When SO_2_ was removed from the stream, the catalyst was able to recover completely. The crystal structure, morphology, textural properties and valence state of the metals involving the novel catalysts were investigated using X-ray diffraction, N_2_ adsorption and desorption analysis, X-ray photoelectron spectroscopy, scanning electron microscopy and energy dispersive spectroscopy, respectively. The decrease of NH_3_–SCR performance in the presence of 100 ppm SO_2_ was due to the decrease of the surface area, change of the pore structure, the decrease of Ce^4+^ and Mn^4+^ concentration and the formation of the sulfur phase chemicals which blocked the active sites and changed the valence status of the elements.

## 1. Introduction

Nitrogen oxides NO*_x_* (NO, NO_2_, N_2_O) are global by-products of high-temperature combustion [1]. NO*_x_* abatement from emissions is necessary because emitted nitrogen oxides cause severe adverse health effects, acid rain and ozone layer depletion [2–6]. Among the NO*_x_* treatment approaches, the technology involving the selective catalytic reduction (SCR) of NO*_x_* with NH_3_ (NH_3_–SCR) has been regarded as the most effective and widely used method because of its high denitration efficiency and wide operable temperature range [7]. The SCR method refers to the process in which the reducing agent NH_3_ reacts with NO*_x_* with the assistance of catalyst, generating N_2_ and H_2_O [8]. The reactions involved in this process can be seen in the following equations

1.1 $$4\text{N}{\text{H}}_{3}+4\text{NO}+{\text{O}}_{2}\to 4{\text{N}}_{2}+6{\text{H}}_{2}\text{O, }$$

1.2 $$4\text{N}{\text{H}}_{3}+2\text{N}{\text{O}}_{2}+{\text{O}}_{2}\to 3{\text{N}}_{2}+6{\text{H}}_{2}\text{O, }$$

1.3 $$4\text{N}{\text{H}}_{3}+3{\text{O}}_{2}\to 2{\text{N}}_{2}+6{\text{H}}_{2}\text{O, }$$

1.4 $$2\text{N}{\text{H}}_{3}+2{\text{O}}_{2}\to {\text{N}}_{2}\text{O}+3{\text{H}}_{2}\text{O}$$

1.5 $$\text{and}\;\;2\text{N}{\text{H}}_{3}+2\text{N}{\text{O}}_{2}+2\text{NO}\to 2{\text{N}}_{2}+3{\text{H}}_{2}\text{O}.$$

Reactions (1.1) and (1.2) are designated as the main reactions, and reaction (1.1) is often regarded as the standard SCR reaction. Elevated temperature may lead to the occurrence of NH_3_ oxidation side effects, partly shown in equations (1.3) and (1.4). The coexistence of NH_3_, NO and NO_2_ will lead to a rapid SCR reaction, shown in equation (1.5). Studies have shown that the reaction rate of equation (1.5) is faster than that of standard SCR reactions.

The fundamental goal of NH_3_–SCR technology is to develop a SCR catalyst that possesses a high activity at relatively low temperature ranges, strong anti-sulfur performance and vanadium-free to be environmentally friendly. Metal oxide catalyst groups that are Mn-based [9–11] and Ce-based [12–14] have high efficiencies at low temperature ranges because of the reduction of Mn^4+^ to Mn^3+^ in the Mn phase, and CeO_2_ has a considerably large oxygen storage capacity and excellent redox properties. Composite catalysts prepared using these two kinds of active components have favourable low-temperature denitration activity and are stable when introduced to typical catalyst poisons. In addition, the vast surface area, developed pore structure and acid sites of TiO_2_ are more conducive to the adsorption of NH_3_ and can accelerate the reaction [15–17]. Qi *et al*. [18] prepared a non-load-type MnO*_x_*-CeO_2_, low-temperature SCR catalyst using co-precipitation. The removal of NO was boosted by improving the ability of the redox catalyst by infusing Mn in the CeO_2_ lattice. This generated a large number of oxygen vacancies. Lee *et al*. [19] prepared a MnO*_x_*/CeO_2_-TiO_2_ catalyst. They found that Ce doping increased the surface area of the catalyst, improved the Mn^4+^ concentration and increased the overall catalytic activity. Liu *et al*. [3] proved that the environmentally benign Mn-Ce-Ti catalyst had a high affinity for NO*_x_* removal because of the dual redox properties and the amorphous structure of the catalyst. Moreover, the Mn-Ce-Ti catalyst displayed a high resistance towards H_2_O and SO_2_.

The principal objective of this study involves determining how well the co-doped Ce-Mn/TiO_2_ catalyst can tolerate sulfur while undergoing low-temperature SCR. A series of Ce-Mn/TiO_2_ catalysts were prepared using a single impregnation (IP) method and investigated for their effectiveness of low-temperature SCR of NO*_x_* with NH_3_. The possible mechanism of the best performing, low-temperature SCR catalyst in this work is discussed in detail using various characterization methods.

## 2. Material and methods

### 2.1. Catalyst preparation

Catalysts were prepared using previously discussed IP and sol-gel (SG) methods [20,21]. The IP method involved using a molar ratio of 2 : 3 manganese acetate to cerium nitrate. The components were dissolved in deionized water and stirred for 1** **h. Subsequently, approximately 2.5** **g of TiO_2_ was added to the mixture and stirred for 2** **h. The suspension was transferred to a rotary evaporator and heated at 55°C for 1** **h to obtain the viscous liquid. This sample was dried at 105°C overnight in an oven. Subsequently, the sample was calcined at 550°C for 6** **h and ground to a mesh size of 20–40. The mass ratio of Ce/TiO_2_ was tuned and recorded as Ce(wt%)-Mn/TiO_2._ The molar ratio of Mn and Ce element maintains constant in each catalyst.

The SG method involved using 2.65** **g of tetra-n-butyl titanate and 21.2** **g of ethanol. The components were mixed and stirred for 50** **min to form a yellow-tinted solution referred to as ‘solution A’. A separate solution of manganese nitrate and cerium nitrate was dissolved in deionized water, and then anhydrous ethanol and acetic acid were added. This solution was stirred for 1** **h to obtain a red-tinted solution referred to as ‘solution B’. Subsequently, solution B was slowly added to solution A, and the mixture was stirred for 12** **h. The gel formed at a room temperature for 2** **h. The gel was then dried at 105°C for 24** **h and calcined at 500°C for 2** **h in a 20% O_2_ flow. The final sample was ground to a mesh size ranging from 20 to 40. The ratio of tetra-n-butyl titanate, ethanol, deionized water and acetic acid was 1 : 8 : 6 : 3. The molar ratio of manganese element and cerium was 2 : 3 (the same ratio used with the IP method outlined above).

### 2.2. Catalyst activity measurement

SCR activity of the catalyst was measured using a fixed bed, stainless steel tube reactor with an inner diameter of 11** **mm and an outer diameter of 14** **mm. Gas purchased to be used in the experiment contained specific concentrations of components to simulate flue gas. The feed gas mixture consisted of 500** **ppm NH_3_, 500** **ppm NO, 3% O_2_ (volume fraction), 0–200** **ppm SO_2_ (depending on the experiment) and a balance of N_2_. The simulated gas flow rate was 1000 ml min^−1^. A volume of 6 ml of catalyst was loaded into the reactor for each experiment. The temperature ranged 100°C and 300°C at a heating rate of 3°C/min with a gas hourly space velocity (GHSV) of 10 000 h^−^^1^. The concentrations of NO were monitored at the inlet and outlet in real time using a gas analyser (Testo 340) to calculate the conversion rate using the following equation.

2.1 $$\mathrm{NO}\;\mathrm{conversion}\;(\%)=\frac{{\left[\mathrm{NO}\right]}_{\mathrm{in}}-{\left[\mathrm{NO}\right]}_{\mathrm{out}}}{{\left[\mathrm{NO}\right]}_{\mathrm{in}}}\times 100\%,$$

where [NO]_in_ and [NO]_out_ referred to the NO concentration at the reactor inlet and outlet, respectively. The concentration was measured when the reaction reached a steady-state condition (approx. 20–40** **min) at each temperature, which reduced the measurement errors caused by instability.

### 2.3. Catalyst characterization

The powder X-ray diffraction (XRD) characterization of the samples was performed using a Bruker D8-Advance X-ray powder diffractometer with a Cu K*α* radiation source (*λ* = 1.5406 Å), a pulverized sample with scattering angles (2*θ*) of 5–85° and a 0.0197 step size operated at 50** **kV and 50** **mA. The diffraction lines were identified by matching them with reference patterns in the Joint Committee on Powder Diffraction Standards (JCPDS) database.

A ThermoFisher Escalab 250Xi X-ray powder photoelectron spectrometer was used to qualitatively analyse the X-ray photoelectron spectroscopy (XPS) characterization of the sample surface composition using an Al K*α* radiation source with a scattering of 0–5000** **eV. The binding energy was calibrated using the C 1s peak contaminate carbon (BE = 284.6** **eV) as an internal standard.

N_2_ adsorption and desorption of each sample were measured at −196°C using the ASAP 2020 automatic rapid surface area and mesoporous/microporous analyser with a N_2_ adsorption gas. The samples were degassed at 200°C for 12** **h before the measurement took place. The specific surface area was calculated according to the Brunauer–Emmett–Teller (BET) method. The total pore volume was determined based on the amount of the adsorbed N_2_ volume at a relative pressure of approximately *p*/*p*_0_ = 0.99.

The surface morphology of the catalysts was characterized with scanning electron microscopy (SEM) using a Hitachi SU8010 with an acceleration voltage of 15** **kV. Qualitative and quantitative analysis of catalyst elements were carried out using energy dispersive X-ray spectroscopy (EDS) on a HORIBA EX-350 with an acceleration voltage of 15 KV.

## 3. Results

### 3.1. Effect of synthetic method on the NH_3_–SCR activity

**Figure 1.**
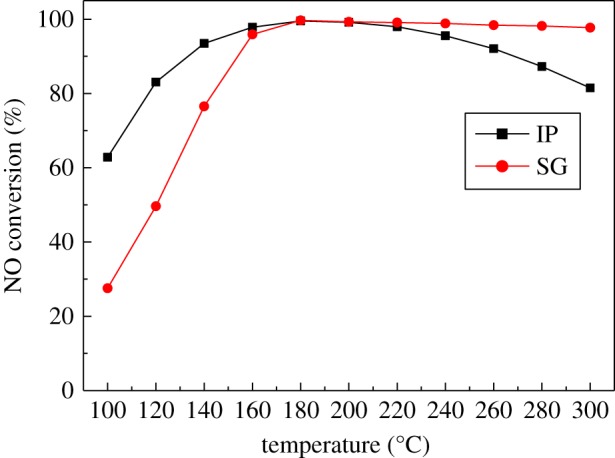
The influence of synthesis methods on NH_3_–SCR activity. IP, impregnation method; SG, sol-gel method (500 ppm NO, 500 ppm NH_3_, 3% O_2_, N_2_ balance gas, GHSV = 10 000 h^−1^).

SCR activities of the Ce(20)-Mn/TiO_2_ catalysts synthesized by the SG and IP methods are shown in figure 1. These results reveal that the IP method had a better low-temperature denitration activity than the SG method. At a temperature range of 140–260°C, the NO conversion rate was higher than 90% in the presence of the IP catalyst. At a temperature range of 160–300°C, the NO conversion rate was higher than 90% in the presence of the SG catalyst. The NO conversion rate was approximately 99.7% at a temperature of 180°C using either method. The catalyst made using the IP method showed better low-temperature denitration activity, was easy to prepare, and had a low-cost preparation process. Therefore, the following Ce-Mn/TiO_2_ catalysts used in this study were prepared using the IP method.

### 3.2. Resistance to SO_2_ poisoning on Ce-Mn/TiO_2_ catalysts

**Figure 2.**
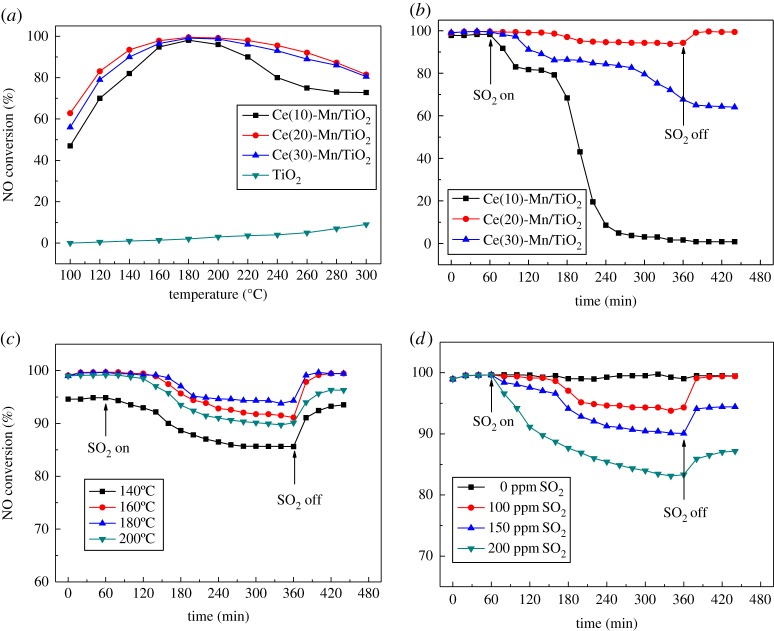
(*a*) Catalytic activity of Ce-Mn/TiO_2_ catalyst for NH_3_–SCR. Catalysts were loaded with 10%, 20% and 30% Ce and denoted as Ce(10), Ce(20) and Ce(30), respectively. Pure TiO_2_ was also used for comparison. (*b*) The effect of various Ce concentrations using a Ce-Mn/TiO_2_ catalyst on SO_2_ resistance. (*c*) The effects of reaction temperature on NO conversion of the Ce(20)-Mn/TiO_2_ catalyst in the presence of SO_2_. The above three types of reactions were performed at: 500** **ppm NO, 500** **ppm NH_3_, SO_2_ 100** **ppm, 3% O_2_, N_2_ balance gas, GHSV = 10 000 h**^−^**^1^. (*d*) The effects of SO_2_ concentration on NO conversion of Ce(20)-Mn/TiO_2_ catalysts (*T* = 180°C, 500** **ppm NO, 500** **ppm NH_3_, 3% O_2_, N_2_ balance gas, GHSV = 10 000 h**^−^**^1^).

The addition of active components has a crucial effect on the catalyst performance in NH_3_–SCR. As shown in figure 2*a*, the NO*_x_* conversion rate on pure TiO_2_ was less than 10% in the temperature range of 100–300°C, indicating that the carrier was inactive for denitration. After adding Ce and Mn, the catalytic activity of Ce-Mn/TiO_2_ was enhanced substantially. Catalysts were loaded with 10%, 20% and 30% Ce and denoted as Ce(10), Ce(20) and Ce(30), respectively. The temperature windows for obtaining a denitration rate higher than 90% included 160–220°C for the Ce(10)-Mn/TiO_2_ catalyst, 140–260°C for the Ce(20)-Mn/TiO_2_ catalyst and 160–240°C for the Ce(30)-Mn/TiO_2_ catalyst. Therefore, the Ce(20)-Mn/TiO_2_ catalyst had the highest NO conversion rate of 99.7% with a wide denitration low-temperature window.

In the absence of SO_2_, all three Ce-Mn/TiO_2_ catalysts had a NO conversion of approximately 100% (during the initial 1 h shown in figure 2*b*). After adding 100 ppm of SO_2_, the NH_3_–SCR activity of Ce(10)-Mn/TiO_2_ and Ce(30)-Mn/TiO_2_ decreased rapidly to 0% and 68% in 5 h, respectively. After SO_2_ was removed from the stream, the Ce(10)-Mn/TiO_2_ catalyst lost all of its activity, while the Ce(30)-Mn/TiO_2_ catalyst only maintained an activity of approximately 65%. However, the Ce(20)-Mn/TiO_2_ catalyst had only a small decrease of 6% in the NO conversion after 100 ppm of SO_2_ was added in 5 h. After SO_2_ was removed from the stream, the NH_3_–SCR activity involving the Ce(20)-Mn/TiO_2_ catalyst recovered completely and reached 100% NO conversion.

The effect of reaction temperature on the sulfur resistance of the Ce(20)-Mn/TiO_2_ catalyst was investigated, and the results are shown in figure 2*c*. The addition of SO_2_ at various reaction temperatures all had an inhibitory effect on the catalytic activity. The conversion rate of NO constantly decreased with the addition of 100 ppm SO_2_. The decrease in catalytic activity at 140°C was the most pronounced, while the catalyst activity at 180°C had the best sulfur resistance.

The effect of the SO_2_ concentration on the activity of the best Ce(20)-Mn/TiO_2_ catalyst was investigated, and the results are shown in figure 2*d*. When the catalyst was at 180°C for 1 h, the addition of SO_2_ in different concentrations decreased the catalytic activity, and there were different levels of recovery after SO_2_ was removed from the stream. SO_2_ had a toxic effect on the Ce(20)-Mn/TiO_2_ catalyst, and as the concentration of SO_2_ increased, the catalyst activity decreased. The addition of 100, 150 and 200 ppm SO_2_ decreased the NO conversion rate from 99.7% to 94%, 90% and 83%, respectively. After removing 100 ppm of SO_2_, the catalyst activity fully recovered; however, only partial recovery was seen after the catalyst was submitted to the higher SO_2_ concentrations.

### 3.3. Catalyst characterization

**Figure 3.**
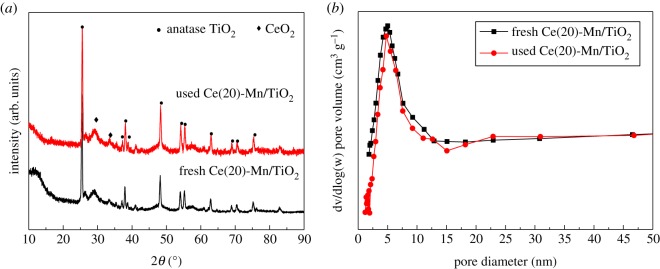
(*a*) XRD patterns and (*b*) pore size distribution of fresh and used Ce(20)-Mn/TiO_2_ catalysts.

The Ce(20)-Mn/TiO_2_ catalyst had the best NH_3_–SCR performance, so only fresh and used Ce(20)-Mn/TiO_2_ catalyst were analysed for determining specific catalyst characteristics. The used catalyst was extracted after the reaction was carried out in a 100** **ppm SO_2_ atmosphere for 5** **h at 180°C. The XRD patterns in figure 3*a* showed that no new peaks were observed in the used catalyst, indicating that the catalyst did not form any (or negligible) crystalline states of sulfate or the amount of the formed phase was too small in the SO_2_ atmosphere. Based on the activity discussion mentioned above, the NO conversion rate of the Ce(20)-Mn/TiO_2_ catalyst was approximately 94% after the addition of 100** **ppm SO_2_. Therefore, the Ce(20)-Mn/TiO_2_ catalyst had an ideal sulfur resistance.

The specific surface area, pore volume and average pore size of the different Ce(wt%)-Mn/TiO_2_ catalysts are summarized in table 1. Owing to the large specific surface area of CeO_2_, the loading amount of Ce had a significant impact on the specific surface area of the catalyst. With the increase in the loading amount, the specific surface area showed a tendency to increase first and then decrease. The increase in the loading amount of Ce led to a discernable change of the specific surface area from 23.822 to 35.024 m^2^ g^−1^ and finally to 34.839 m^2^ g^−1^, indicating that the doping of Ce played an important role in the variation of the specific surface area of the catalyst. The surface area of Ce(10)-Mn/TiO_2_ is relatively small, which corresponds to the lowest catalytic activity of Ce(10)-Mn/TiO_2_ in figure 2*a*.

**Table 1.** Physical properties of Ce(wt%)-Mn/TiO_2._

catalyst	specific surface area (m^2^ g^−1^)	pore volume (cm^3^ g^−1^)	average pore size (nm)
Ce(10)-Mn/TiO_2_	23.822	0.0575	11.16
Ce(20)-Mn/TiO_2_	35.024	0.0631	8.09
Ce(30)-Mn/TiO_2_	34.839	0.0504	5.06

The specific surface area, pore volume and average pore size of the fresh and used catalyst are summarized in Table 2. The physical properties of the catalyst were impaired by SO_2_. The addition of 100** **ppm SO_2_ decreased the respective specific surface area, pore volume and average pore size of the Ce(20)-Mn/TiO_2_ catalyst from 35.024 m^2^ g^−1^, 0.0631** **cm^3^ g^−1^ and 8.09** **nm to 28.092 m^2^ g^−1^, 0.0624** **cm^3^ g^−1^ and 6.15** **nm, respectively. The pore size distribution of fresh and used Ce(20)-Mn/TiO_2_ catalysts are shown in figure 3*b*. The pore structure of the fresh Ce(20)-Mn/TiO_2_ catalyst was in the range of 2–10** **nm. After the reaction, the number of pores in the range of 8–10** **nm decreased slightly, while the number of pores in the 2–8** **nm range did not change, ensuring there was sufficient catalytic activity. The decreased NO conversion was due to the change in the catalyst structure in terms of the pore size distribution and surface area.

**Table 2.** Physical properties of fresh and used Ce(20)-Mn/TiO_2_ catalyst.

catalyst	specific surface area (m^2^ g^−1^)	pore volume (cm^3^ g^−1^)	average pore size (nm)
fresh Ce(20)-Mn/TiO_2_	35.024	0.0631	8.09
used Ce(20)-Mn/TiO_2_	28.092	0.0624	6.15

**Figure 4.**
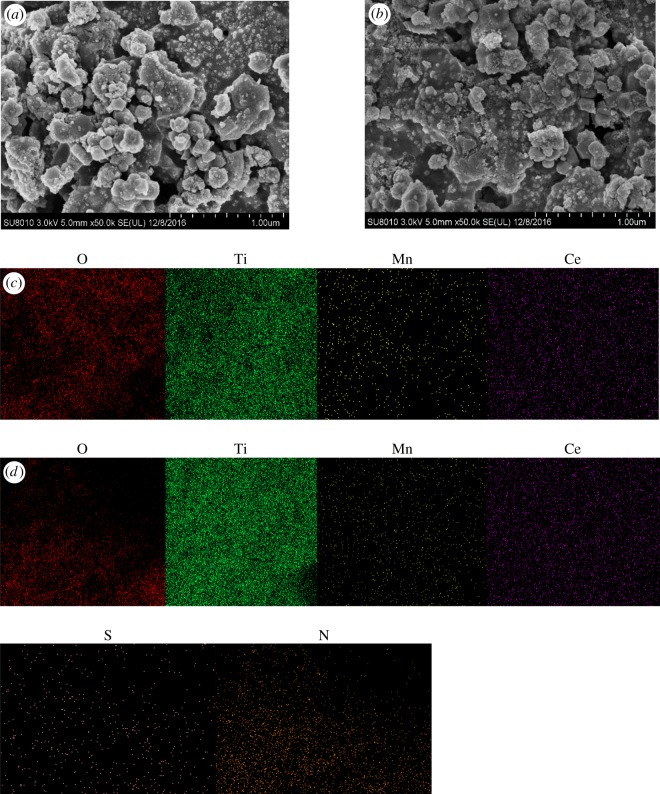
SEM micrographs of (*a*) fresh and (*b*) used Ce(20)-Mn/TiO_2_ catalysts as well as energy dispersive spectrometer images of (*c*) fresh and (*d*) used Ce(20)-Mn/TiO_2_ catalyst.

The fresh and used Ce(20)-Mn/TiO_2_ catalysts were also characterized by SEM-EDS, and the results are shown in figure 4. An agglomeration phenomenon, though not obvious, occurred on the surface of the catalyst after undergoing reactions in the SO_2_ atmosphere (figure 4*a–b*). EDS mapping results (figure 4*c–d* and table 3) verified the existence of S and N on the surface, implying that the ammonium sulfate material formed on the surface of the used catalyst. This covered the active sites and decreased the catalytic activity. However, the XRD patterns imply that the ammonium sulfate material was not obviously detected because there is no formation or only a relatively small amount was present and therefore accounted for only a negligible decrease of the catalytic activity in the SO_2_ atmosphere.

**Table 3.** Surface element concentration of fresh and used Ce(20)-Mn/TiO_2_ catalyst.

	the concentration of surface elements (%)					
catalyst	Ce	Mn	O	Ti	S	N
fresh Ce(20)-Mn/TiO_2_	10.37	8.96	75.22	5.45	—	—
used Ce(20)-Mn/TiO_2_	10.1	8.01	71.41	5.23	3.28	1.97

**Figure 5.**
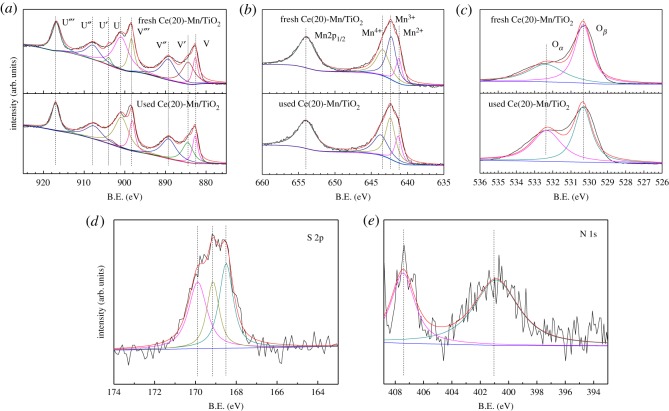
XPS spectra of fresh and used Ce(20)-Mn/TiO_2_. (*a*) Ce 3d, (*b*) Mn 2p, (*c*) O 1s, (*d*) S 2p and (*e*) N 1s.

XPS spectra of the Ce 3d, Mn 2p and O 1s of the fresh and used Ce(20)-Mn/TiO_2_ catalysts are shown in figure 5*a–c*. Figure 5*a* shows the Ce 3d in the fresh and used catalysts had eight peaks, where the V′, U′ were the characteristic peaks of Ce^3+^ (Ce_2_O_3_), and V, V^″^, V^‴^, U, U^″^, U^‴^ were the characteristic peaks of Ce^4+^. The leading form of the Ce oxide in both catalysts was CeO_2_ (Ce^4 +^) which was beneficial for oxygen storage capacity. The conversion between Ce^3+^ and Ce^4+^ accomplished a storage-release cycle of oxygen for better promoting the process of denitration. Figure 5*b* illustrates that the peak of Mn 2p_1/2_ was located near 653.3** **eV, while the peak of Mn 2p_3/2_ was composed of Mn^2+^, Mn^3+^ (642.0** **eV) and Mn^4+^ (643.0** **eV). The leading form of Mn in the Ce(20)-Mn/TiO_2_ catalyst was Mn^4+^ (table 4). The catalytic effect of MnO*_x_* was related to its valence state, where MnO_2_>Mn_2_O_3_>MnO [22]. This has also been demonstrated by Thirupathi & Smirniotis [23]. Their group indicated that MnO_2_ was the most active component among a series comprising MnO_2_, Mn_5_O_8_, Mn_2_O_3_ and Mn_3_O_4_. Therefore, Mn^4+^ had a superior catalytic denitration activity, and this corresponded to the best catalytic denitration activity of Ce(20)-Mn/TiO_2_. The peaks of the Mn 2p_1/2_ and Mn 2p_3/2_ of the used catalyst shifted in the direction of a higher electron bonding energy, and the offsets were approximately 0.06** **eV and 0.1** **eV, respectively. This indicated that a metal sulfate formed which resulted from the sulfation of Mn [24]. The decrease of Mn^4+^/Mn^3+^ from 84.4% to 75.4% is a possible reason for the decreased NH_3_–SCR activity. The spectrum of O 1s in figure 5*c* contains characteristic peaks of O*_α_* and O*_β_*, where O*_β_* belongs to the characteristic peak of lattice oxygen at a binding energy of 529.5–529.8** **eV. O*_α_* belongs to the surface adsorption characteristic peaks of oxygen at a binding energy of 531.8–532.5** **eV [10]. In table 4, the surface adsorption of the oxygen concentration was 48.8% in the fresh catalyst, and this increased to 40.5% in the used catalyst. The high concentration of the surface adsorption of oxygen had a strong oxidation effect, which not only completed the oxidation and reduction cycle but also enhanced the oxidation process of NO to NO_2_. This promoted a rapid response to the SCR reaction [25,26].

**Table 4.** Surface elemental valence distribution of fresh and used Ce(20)-Mn/TiO_2_ catalyst.

	the valence ratio of the surface elements (%)		
catalyst	Ce^3+^/Ce^4+^	Mn^4+^/Mn^3+^	O*_α_*/(O*_α_*+O*_β_*)
fresh Ce (20)-Mn/TiO_2_	17.5	84.4	48.8
used Ce (20)-Mn/TiO_2_	18.0	75.3	40.5

XPS spectra of the S 2p and N 1s of the used Ce(20)-Mn/TiO_2_ catalysts are shown in figure 5*d–e*. Figure 5*d* shows that the leading form of S in the used catalyst was S^4+^ and S^6+^. The peak at 168.5** **eV belonged to SO_3_^2−^, while the peaks at 169.9 and 169.1** **eV were assigned to SO_4_^2−^ [24]. S^6+^ accounted for approximately 63% of S and included some sulfate material which formed on the catalyst surface after undergoing reactions in the SO_2_ atmosphere. Figure 5*e* demonstrates the peaks of N located in the vicinity of 400.8** **eV and 407.3** **eV were characterized as NH_4_^+^ and NO_3_^−^, respectively [27]. NH_4_^+^ accounted for approximately 64% of the total N. The conversion of NH_3_ to NH_4_^+^ and NO_3_^−^ was indicated by the results.

## 4. Conclusion

NH_3_–SCR activity was measured after using an IP method and a SG method to form a Ce-Mn/TiO_2_ catalyst. The catalyst formed with the IP method had a better low-temperature denitration activity than the catalyst synthesized using the SG method. The Ce-Mn/TiO_2_ catalyst loaded with 20% Ce had the best low-temperature activity with a NO conversion rate of 99.7% at 180°C. Despite the addition of 100** **ppm SO_2_ at 180°C for 5** **h, the NO conversion rate of the Ce(20)-Mn/TiO_2_ catalyst was still as high as 94% and recovered when SO_2_ was removed from the stream. The SO_2_ resistance of the Ce(20)-Mn/TiO_2_ catalyst is attributed to the widely distributed elements of Mn and Ce. This led to the inability of the sulfate material to remain on the surface. Characterization of fresh and used Ce(20)-Mn/TiO_2_ catalysts was performed using XRD, BET, XPS and SEM-EDS. These results indicated that there was a decrease in Ce^4+^ and Mn^4+^ in the used catalysts. These results also showed that the sulfate phase formation and the change of the pore structure accounted for a decrease in NH_3_–SCR activity.

## Data, accessibility

Our data are deposited at Dryad: https://doi.org/10.5061/dryad.pv12j [28].
